# Sex-Specific Effects of *NLRP6/AVR* and *ADM* Loci on Susceptibility to Essential Hypertension in a Sardinian Population

**DOI:** 10.1371/journal.pone.0077562

**Published:** 2013-10-11

**Authors:** Nicola Glorioso, Victoria L. Herrera, Tamara Didishvili, Maria F. Ortu, Roberta Zaninello, Giovanni Fresu, Guiseppe Argiolas, Chiara Troffa, Nelson Ruiz-Opazo

**Affiliations:** 1 Hypertension and Related Diseases Center, AUO-Universita’ di Sassari, Sassari, Sardinia, Italy; 2 Department of Medicine, Boston University School of Medicine, Boston, Massachusetts, United States of America; Instituto de Higiene e Medicina Tropical, Portugal

## Abstract

Coronary artery disease, heart failure, fatal arrhythmias, stroke, and renal disease are the most common causes of mortality for humans, and essential hypertension remains a major risk factor. Elucidation of susceptibility loci for essential hypertension has been difficult because of its complex, multifactorial nature involving genetic, environmental, and sex- and age-dependent nature. We investigated whether the 11p15.5 region syntenic to rat chromosome 1 region containing multiple blood pressure quantitative trait loci (QTL) detected in Dahl rat intercrosses harbors polymorphisms that contribute to susceptibility/resistance to essential hypertension in a Sardinian population. Initial testing performed using microsatellite markers spanning 18 Mb of 11p15.5 detected a strong association between D11S1318 (at 2.1 Mb, P = 0.004) and D11S1346 (at 10.6 Mb, P = 0.00000004), suggesting that loci in close proximity to these markers may contribute to susceptibility in our Sardinian cohort. NLR family, pyrin domain containing 6/angiotensin-vasopressin receptor (*NLRP6/AVR*), and adrenomedullin (*ADM*) are in close proximity to D11S1318 and D11S1346, respectively; thus we tested single nucleotide polymorphisms (SNPs) within *NLRP6/AVR* and *ADM* for their association with hypertension in our Sardinian cohort. Upon sex stratification, we detected one *NLRP6/AVR* SNP associated with decreased susceptibility to hypertension in males (rs7948797G, *P* = 0.029; OR = 0.73 [0.57–0.94]). For *ADM*, sex-specific analysis showed a significant association between rs4444073C, with increased susceptibility to essential hypertension only in the male population (*P* = 0.006; OR = 1.44 [1.13–1.84]). Our results revealed an association between *NLRP6/AVR* and *ADM* loci with male essential hypertension, suggesting the existence of sex-specific *NLRP6/AVR* and *ADM* variants affecting male susceptibility to essential hypertension.

## Introduction

Essential hypertension is a highly prevalent disorder and remains a major risk factor for the most common causes of mortality, including coronary artery disease, heart failure, fatal arrhythmias, stroke, and renal disease [1,2]. As a complex, multifactorial disorder, elucidation of susceptibility loci remains difficult. Previous studies have emphasized the challenges of genetically analyzing essential hypertension [3-5]. Genome-wide association studies have failed to detect major hypertension susceptibility genes that may contribute greater than 5 mmHg to the blood pressure (BP) effect [6-9]. These studies reported that several loci were significantly associated with increased BP by analyzing tens of thousands of patients in a multi-center, meta-analysis paradigm with BP effects ranging from 0.5–1 mmHg [6-9]. This implies that either hundreds of hypertension susceptibility genes exist that account for essential hypertension in humans, with each locus contributing a small fraction of the increase in BP (0.5–1 mmHg effect), or the studies have failed to detect major hypertension susceptibility loci due to major confounders, such as inherent genetic heterogeneity of human populations, great variability in trait measurements, the existence of several factors that are not accounted for, and factors in the analytical paradigm (e.g., sex-specific effects, gestational risk factors, and presence of hypertension subtypes with unique pathogenetic mechanisms). 

For complex multifactorial diseases with clinical heterogeneity such as hypertension, genetic studies of inbred rat models of polygenic (essential) hypertension are instrumental for identifying BP-quantitative trait loci (QTL) and candidate susceptibility genes for subsequent testing in human essential hypertension. We have successfully employed this approach to identify *ATP1A1* (α1 Na,K-ATPase) [10,11] and *DEspR* (dual endothelin1/vascular endothelial growth factor signal peptide receptor) [12,13] as candidate hypertension genes in a Dahl salt-sensitive rat model of polygenic hypertension. Subsequent studies detected an association of *ATP1A1* [14,15] and *DEspR* [15] with essential hypertension in humans and revealed a functionally significant *DEspR* T/CATAAAA-box promoter variant associated with 7.7 mmHg in increased systolic BP in a male Sardinian population [16]. We recently reported two closely-linked BP QTLs on chromosome-1 (chr1) (*BP-m1* at 144.3 Mb and *BP-m2* at 208.8 Mb) affecting salt-sensitive hypertension in Dahl rats [12]. These two chr1-BP QTLs contain some candidate genes, which has been supported by experimental evidence. Briefly, molecular genetic evidence demonstrates that a functionally significant N119S/C163R variant of the angiotensin-vasopressin receptor (*NLRP6/AVR* at 201.08 Mb), which exhibits sodium-induced dysfunction [17], may underlie chr1 *BP-m1* QTL of salt-sensitive hypertension in F2 (Dahl S x Dahl R]-intercross male rats [12]. Another candidate gene in this chr1-BP QTL region includes adrenomedullin (*ADM*; at 168.38 Mb), which has been implicated in hypertension pathogenesis [18,19] and may underlie *BP-m2* QTL [12]. 

Analyses of *BP-m1* and *BP-m2* corresponding syntenic regions in humans localized this chromosomal segment to the 11p15.5 region. We therefore investigated whether this 11p15.5 region harbors polymorphisms contributing to susceptibility/resistance to essential hypertension in our Sardinian population.

## Results

### Association between 11p15.1-11p15.5 region and essential hypertension

To enhance robustness of the population under study, we ascertained first a limited genetic diversity by restricting the cohort under analysis to a relatively isolated genetic population of northern Sardinia [20,21] and second , we minimized subtype heterogeneity by focusing on the extreme of the population to distinguish hypertensive patients and normotensive controls. Thus, we compared ascertained hypertensive patients (n = 433) with group means for systolic BP (SBP) of 174.4 ± 14.7 mmHg and diastolic BP (DBP) of 110.5 ± 9.9 mmHg against control normotensives (n = 279) with group means for SBP of 127.6 ± 11.3 mmHg and DBP of 77.6 ± 7.2 mmHg (Table 1). Both groups contained equivalent representation of both sexes (Table 1). 

**Table 1:** Characteristics of the study population.

**Variable**	**NT^a^ (total)**	**HT^b^ (total)**	**Male NT**	**Female NT**	**Male HT**	**Female HT**
n	279	433	131	148	237	196
Age, y^c^	65.4 ± 10.6	51.0 ± 10.2	66.1 ± 8.9	64.8 ± 11.9	51.8 ± 10.6	50.0 ± 9.6
BMI^d^, Kg^e^/m^2^	26.2 ± 3.9	27.7 ± 4.0	26.3 ± 3.0	26.2 ± 4.6	28.0 ± 3.8	27.4 ± 4.3
SBP^f^, mmHg^g^	127.6 ± 11.3	174.4 ± 14.7	127.9 ± 10.7	127.4 ± 11.9	173.2 ± 14.6	175.9 ± 14.8
DBP^h^, mmHg	77.6 ± 7.2	110.5 ± 9.9	77.2 ± 6.8	78.0 ± 7.4	111.9 ± 10.4	108.8 ± 9.0

^a^ Normotensives; ^b^ hypertensives; total, male + female subjects; ^c^ years; ^d^ body mass index; ^e^ kilogram per meter squared; ^f^ systolic blood pressure; ^g^ millimeters mercury; ^h^ diastolic blood pressure.

Initial testing of possible loci associated with essential hypertension within the 11p15.1–11p15.5 region was performed with microsatellite markers spanning 18 Mb of this chromosome 11 region. Since microsatellite markers are highly variable, they typically reveal a significant amount of information; therefore, they may be useful for examining large genomic segments in linkage and/or association studies [22,23]. As shown in Table 2, the D11S1318 and D11S1346 microsatellite markers showed a strong association with hypertension susceptibility after adjusting for multiple testing (D11S1318, *P* = 0.004; D11S1346, *P* = 0.00000004). This suggests that loci in closed proximity to D11S1318 and D11S1346 contribute to hypertension susceptibility in our Sardinian cohort.

**Table 2:** Association analysis of 11p15.1-11p15.5 region with hypertension susceptibility.

Marker	Position	χ^2^	*df*	*P*	*P_b_*	Candidate (bp)
D11S1318	2327223	27.17	8	0.00066	**0.0040**	*NLRP6/AVR* (278570)
D11S1338	5987976	4.025	2	0.1337	0.8022	
D11S1323	6276714	2.914	3	0.4050	1.0000	
D11S1346	10959374	43.74	4	0.000000007	**0.00000004**	*ADM* (10327248)
D11S1315	12804576	1.508	4	0.8253	1.0000	
D11S1310	17622865	1.755	2	0.4158	1.0000	

*NLRP6,NLR family, pyrin domain containing 6*; *AVR*, angiotensin-vasopressin receptor; *ADM*, adrenomedullin. *df*, degrees of freedom; *P*, nominal *P* values; *P*_*b*_, *P* values with the Bonferroni adjustment. Significant results after bonferroni adjustment (P < 0.05) are shown in boldface type. Marker positions on chromosome 11p15.1-11p15.5 region as per NCBI Human Annotation Release 104, GRCh37.p10-Primary Assembly.

### Single-point association analysis of *NLRP6/AVR* and *ADM* loci

**Figure 1:**
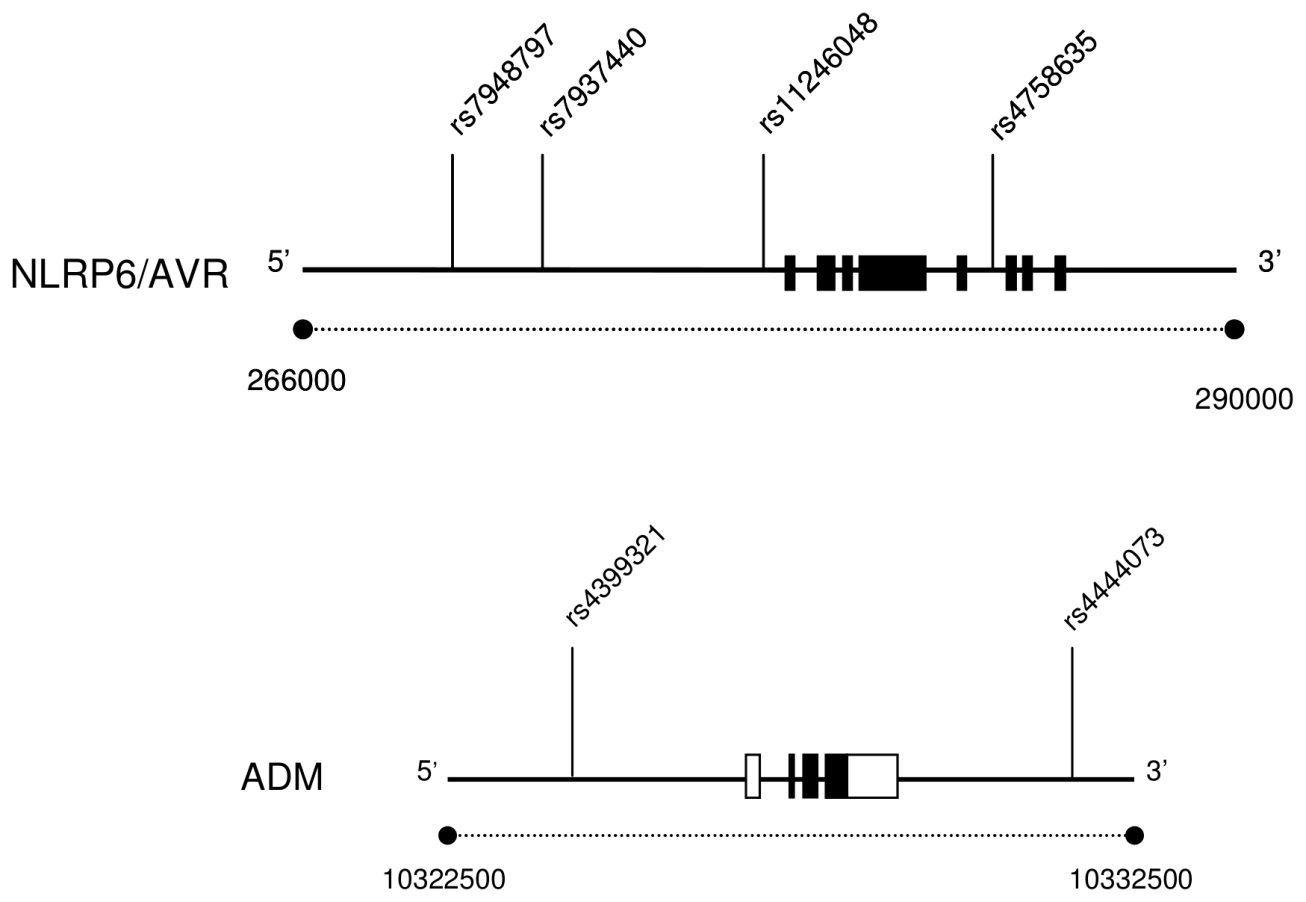
Structure of *NLRP6/AVR* and *ADM* genes and location of the SNPs analyzed. Exons (shown as boxes) 1–8 for *NLRP6/AVR* and exons 1–4 for *ADM* are shown. Gene untranslated (5′-untranslated and 3′-untranslated) regions are unfilled. Corresponding nucleotide positions for the *NLRP6/AVR* and *ADM* loci on chromosome 11 are indicated in bp. Locations of the SNPs genotyped are shown by vertical lines.

Closed examination of genes in the vicinity of D11S1318 and D11S1346 identify *NLRP6/AVR* (at 278570) and *ADM* (at 10327248) as candidates for the corresponding associated regions marked by D11S1318 and D11S1346, respectively (Table 2). To assess the possible association of *NLRP6/AVR* and *ADM* with essential hypertension in our Sardinian cohort, we examined single-point associations between *NLRP6/AVR* and *ADM* SNPs (Figure 1, Table 3) with hypertension susceptibility. We used 4 tagged SNPs for the *NLRP6/AVR* locus and 2 tagged SNPs for the *ADM* locus (see Materials and Methods section). Genomic location, allele frequency, and Hardy-Weinberg test results for the 6 tagged SNPs are presented in Table 3. None of the tagged SNPs deviated significantly from Hardy-Weinberg equilibrium in both hypertensive and normotensive cohorts (Table 3). One of the *NLRP6/AVR* SNPs, rs7937440, demonstrated a significant association with hypertension in the total cohort after adjustment for multiple testing (*P* = 0.032, Table 4). Upon sex stratification, one *NLRP6/AVR* SNP (minor allele rs7948797G, Table 4) was associated with decreased susceptibility to hypertension only in the male population (*P* = 0.029; OR = 0.63 [0.45–0.88], Table 4). For *ADM*, sex-specific analysis detected a significant association between rs4444073C minor allele and increased susceptibility to hypertension exclusively in the male cohort as well (*P* = 0.013; OR = 1.62 [1.14–2.29], Table 4). 

**Table 3:** Description of the tagged SNPs genotyped.

**Gene (chr), SNP**	**A/R**	**Position**	**MAF**	***P* (NT)**	***P* (HT)**
*NLRP6/AVR* (11)					
rs7948797	A/G	269856	0.295	0.603	0.528
rs7937440	A/G	272199	0.417	0.601	0.636
rs11246048	T/C	278039	0.365	0.533	0.238
rs4758635	G/T	283928	0.470	0.438	0.956
*ADM* (11)					
rs4399321	A/G	10323478	0.280	0.239	0.148
rs4444073	A/C	10331664	0.344	0.988	0.572

*NLRP6*, *NLR family, pyrin domain containing 6*; *AVR*, angiotensin-vasopressin receptor; *ADM*, adrenomedullin; A/R, ancestral allele/rare allele; MAF, minor allele frequency; *P* (NT), Hardy-Weinberg *P* value in normotensives; *P* (HT), Hardy-Weinberg *P* value in hypertensives. SNP locations as per NCBI Human Annotation Release 104, GRCh37.p10-Primary Assembly.

**Table 4:** Single-point association analysis of SNPs in NLRP6/AVR and ADM with hypertension.

**Gene (chr)**	**SNP**	**Position**	***P^a^***	**OR (95% c.i.)**	***P^b^***	**OR (95% c.i.)**	***P^c^***	**OR (95% c.i.)**
*NLRP6/AVR* (11)	rs7948797	269,856	0.056	0.73 (0.57-0.94)	**0.029**	**0.63 (0.45-0.88)**	0.845	0.86 (0.60-1.24)
	rs7937440	272,274	**0.032**	0.76 (0.60-0.95)	0.100	0.73 (0.53-1.00)	0.585	0.79 (0.57-1.09)
	rs11246048	278,039	0.254	1.17 (0.92-1.48)	0.180	1.29 (0.92-1.81)	0.694	1.07 (0.77-1.49)
	rs4758635	283,928	0.191	0.86 (0.68-1.08)	0.150	0.79 (0.57-1.09)	0.838	0.92 (0.67-1.28)
*ADM* (11)	rs4399321	10,323,478	0.104	1.23 (0.96-1.58)	0.142	1.31 (0.91-1.87)	0.410	1.16 (0.81-1.66)
	rs4444073	10,331,664	**0.006**	1.44 (1.13-1.84)	**0.013**	**1.62 (1.14-2.29)**	0.286	1.29 (0.92-1.81)

*NLRP6*, *NLR family, pyrin domain containing 6*; *AVR*, angiotensin-vasopressin receptor; *ADM*, adrenomedullin; *P* ^*a*^, *P* in males + females; *P* ^*b*^, *P* in males; *P* ^*c*^, *P* in females. OR, odds ratio; c.i., confidence interval. Association analysis was performed with the HelixTree Genetic Analysis Software using Basic Allelic Tests: D vs d as genetic model. *P*, Chi-Squared FDR (Chi-squared P adjusted for multiple comparisons with False Discovery Rate procedure). Significant results (*P* < 0.05) are shown in boldface type.

## Discussion

Identifying genes underlying susceptibility to essential hypertension has been difficult. Genome-wide association studies have failed to detect significant associations contributing greater than 5 mmHg in blood pressure despite the large number of subjects examined in different studied cohorts [6-9]. A number of reasons may account for these negative findings, including intrinsic genetic heterogeneity of human populations, differential accuracy and/or modality in trait measurements (blood pressure), exclusion of putative sex-specific effects on the phenotype in the analytical paradigm, and gestational risk factors. 

Here, we present evidence suggesting that the *NLRP6/AVR* and *ADM* loci contribute to hypertension susceptibility in a Sardinian population. We first detected strong association of the 11p15.1–11p15.5 region with hypertension showing two distinct peaks marked by D11S1318 and D11S1346 SSLP markers. Their chromosomal location indicated *NLRP6/AVR* and *ADM* as putative loci underlying the observed associations. Subsequent analysis using SNPs spanning both the *NLRP6/AVR* and *ADM* transcription units confirmed their associations with essential hypertension in our Sardinian cohort. Both loci demonstrated sex-specific effects on hypertension susceptibility and were primarily associated with male essential hypertension. 

ADM is a potent vasodilatory peptide important for blood pressure homeostasis, as well as cardiovascular and renal function [18,24,25]. Its role in blood pressure regulation has been substantiated by showing that heterozygous *ADM* ^*+/-*^ knockout male mice exhibited elevated blood pressure when compared with wild-type littermates [26]. The ADM locus has been detected as a genome-wide significant finding in a recently reported study involving a large population [8]. In addition, the ADM SNP rs4399321 utilized in our study has been reported to be associated with proteinuria in subjects with essential hypertension [27] and with BP levels in normotensive subjects in a Chinese population [28] supporting a regulatory role for ADM in blood pressure homeostasis in humans. Moreover, recent studies have implicated ADM in hypertension pathogenesis in humans. A dose-response relationship between ADM and BP status was observed among age- and sex-matched normotensive, pre-hypertensive, and hypertensive subjects, detecting lower ADM levels in the hypertensive group than in the pre-hypertensive and normotensive groups [19]. Moreover, ADM levels were found to be associated with mean arterial pressure in men but not in women in the Framingham cohort, thus indicating sex-specificity [29]. This observation is consistent with our results showing that *ADM* is associated with hypertension susceptibility only in the male Sardinian population. 

*NLRP6/AVR* has been linked to salt-sensitive hypertension in Dhal rats. Genetic analysis in an F2 (Dahl S × Dahl R)-intercross rat population delineated *NLRP6/AVR* as a candidate gene underlying a chromosome 1 *BP-m2* QTL [12]. *BP-m2* has shown sex-specificity since it has been detected only in the F2 male population and not in females [12]. Additionally, functional androgen and estrogen response elements have been delineated within the *NLRP6/AVR* 5′-regulatory region that may contribute to the sex-specific effects of *NLRP6/AVR* [30]. Moreover, an *NLRP6/AVR* structural variant has been identified in Dahl S rats exhibiting sodium-induced dysfunction affecting ligand binding and hormone-dependent signal transduction [17]. This supports a pathogenetic role of *NLRP6/AVR* in salt-sensitive hypertension in Dhal rats. The role of *NLRP6/AVR* in blood pressure regulation is further supported by the recent findings showing that NLRP6/AVR deficiency affects urinary concentrating ability and lowers blood pressure in mice [31]. Our data are consistent with the hypothesis that *NLRP6/AVR* contributes to hypertension susceptibility in humans.

In conclusion, our results revealed an association between *NLRP6/AVR* and *ADM* loci and hypertension susceptibility in a northern Sardinian population, primarily affecting male essential hypertension. Specific *ADM* and *NLRP6/AVR* variants affecting susceptibility to high blood pressure require further investigation. 

## Materials and Methods

### Ethics Statement

This study was performed in strict accordance with the principles expressed in the Declaration of Helsinki. The protocol was approved by the local ethics committee of Local Health Unit-University of Sassari Medical School. Written informed consent was obtained and all clinical investigation was conducted according to the principles expressed in the Declaration of Helsinki. 

### Study population

The study cohort comprised 712 subjects, with 433 patients with essential hypertension and 279 normotensive controls enrolled at the Hypertension Center of the University of Sassari Medical School. All subjects were white, unrelated, born in different domains of North Sardinia previously ascertained to have a high degree of genetic homogeneity [20,21], ascertained to be Sardinian for at least 6 generations, and resided in Sardinia. Hypertensive patients with BP > 160/95 mmHg (n = 433), no secondary hypertension etiology and absence of major comorbid conditions were considered in the study. Older patients were included only if they were diagnosed as hypertensive well before 55 years of age. BP measurements were obtained with patients not taking any medications. Family history of hypertension was investigated, and a complete pedigree was defined. To exclude erroneous control subjects with late-onset hypertension, normotensive controls (n = 279) were limited to those older than 60 years of age who had not been previously diagnosed or treated as hypertensive, had no family history of hypertension and cardiovascular or cerebrovascular disease, and had BP values < 135/85 on at least 4 occasions.

### Identification of tag SNPs in *NLRP6/AVR* and *ADM* transcription units

To identify tag SNPs within the *NLRP6/AVR* and *ADM* transcription units we genotyped 300 random samples from our Sardinian sample with 13 *NLRP6/AVR* (rs7113424, rs10902117, rs17655663, rs7948797, rs7396066, rs7937440, rs7102570, rs10794306, rs11246048, rs4758627, rs4758635, rs3817637, rs741737) and 8 *ADM* (rs73420933, rs4641466, rs4399321, rs3814700, rs5002, rs4698, rs4444073, rs7944706) SNPs and tagged them by using the Carlson Method which is based on the R^2^ LD statistic as implemented by HelixTree Genetic Analysis software. We used the following parameters for tagging markers: 1) Minor Allele Frequency Threshold = 0.1. 2) Linkage Disequilibrium (R^2^) Threshold = 0.8. After Carlson SNP-tagging analysis we selected the following SNPs for association analysis (shown in Table 2): rs7948797, rs7937440, rs11246048, rs4758635 for *NLRP6/AVR* and rs4399321, rs4444073 for *ADM*. 

### Genotyping

Genotyping of microsatellite markers was performed as described previously using 20 ng of DNA [11,14]. Alleles were identified using 6% non-denaturing polyacrylamide gel electrophoresis (1/2× Tris-Borate-EDTA, 750 V for 14 h). Gels were exposed to autoradiograms for genotype readings. SNP genotyping was carried out by the Molecular Genetics Core Facility at the Boston University School of Medicine on a Life Technologies 7900 Real-Time PCR System (Foster City, CA, USA). Chromosomal position of microsatellite markers and single nucleotide polymorphisms (SNPs) were based on the “NCBI build 37.1-Map”. SNP assays (TaqMan assays) were procured from Life Technologies. The genotyping completeness rate was 87%. 

### Statistical analysis

Analysis of microsatellite allele frequencies between cases and controls was performed using χ^2^ analysis (Sigma Plot 11.0) for D11S1318 2x9 χ^2^ analysis (9 alleles), D11S1338 2×3 χ^2^ analysis (3 alleles), D11S1323 2×4 χ^2^ analysis (4 alleles), D11S1346 2×5 χ^2^ analysis (5 alleles), D11S1315 2×5 χ^2^ analysis (5 alleles), and D11S1310 2×3 χ^2^ analysis (3 alleles). Bonferroni adjustment was applied to nominal *P* values for multiple testing corrections. For SNPs, single point association analysis comparing cases and control subjects was conducted using the HelixTree^TM^ SNP & Variation Suite genetic analysis software (version 6.4.3, Golden Helix Inc., Bozeman, MT, USA). A basic allelic test (D vs d) was implemented using a Chi-squared test as statistical method, obtaining as well odds ratios with corresponding confidence limits. Missing genotypes were not included (imputed) in the association analysis. The false discovery rate was utilized for multiple testing corrections. A chi-squared test using our cohort with α = 0.005 predicts statistically significant detection of at least 0.1 in allele frequency differences between groups of 433 hypertensives and 279 normotensive subjects with a Power > 0.8. 
